# Supplementary material for: Dietary Catalase Supplementation Alleviates Deoxynivalenol-Induced Oxidative Stress and Gut Microbiota Dysbiosis in Broiler Chickens
Source: Toxins (Basel). 2022 Nov 28;14(12):830. doi: 10.3390/toxins14120830 (PMC9784562; doi:10.3390/toxins14120830)
Supplement: Supplementary file 1 [file toxins-14-00830-s001.zip › toxins-1953130-supplementary.pdf]

Supplementary Material: Dietary Catalase Supplementation Alleviates Deoxynivalenol-Induced Oxidative Stress and Gut Microbiota Dysbiosis in Broiler Chickens

Weiwei Wang, Jingqiang Zhu, Qingyun Cao, Changming Zhang, Zemin Dong, Dingyuan Feng, Hui Ye * and Jianjun Zuo

Table S1. Composition of the basal diet (air-dry basis)

Ingredients	Content (%)
Corn	54.70
Soybean meal	38.10
Soybean oil	2.60
Limestone	1.20
Dicalcium phosphate	2.00
Sodium chloride	0.26
Lysine	0.01
DL-Methionine (98%)	0.13
Premix[1]	1.00
Total	100.00
Nutrient levels	
Metabolizable energy (MJ/kg)	12.55
Crude protein (%)	21.10
Calcium (%)	1.00
Available phosphorus (%)	0.46
Lysine (%)	1.2
Methionine (%)	0.5

[1]Supplied per kilogram of diet: vitamin A, 5,000 IU; thiamin, 2.5 mg; riboflavin, 15 mg; pyridoxine, 4 mg; vitamin D, 80.75 mg; tocopherol, 31 mg; menadione, 1.6 mg; pantothenic acid, 60 mg; niacin, 15 mg; biotin, 0.5 mg; folic acid, 1.5 mg; choline, 450 mg; Cu, 9.5 mg; Zn, 60 mg; Fe, 70 mg; Mn, 121 mg; Se, 0.45 mg; I, 1.4 mg.

Table S2. The contents of some mycotoxins aside from deoxynivalenol and its acetylated precursors in feed

Items	Content
Zearalenone	0.13 mg/kg
Aflatoxin B1	Less than 0.2 μg/kg
Fumonisins	1.85 mg/kg

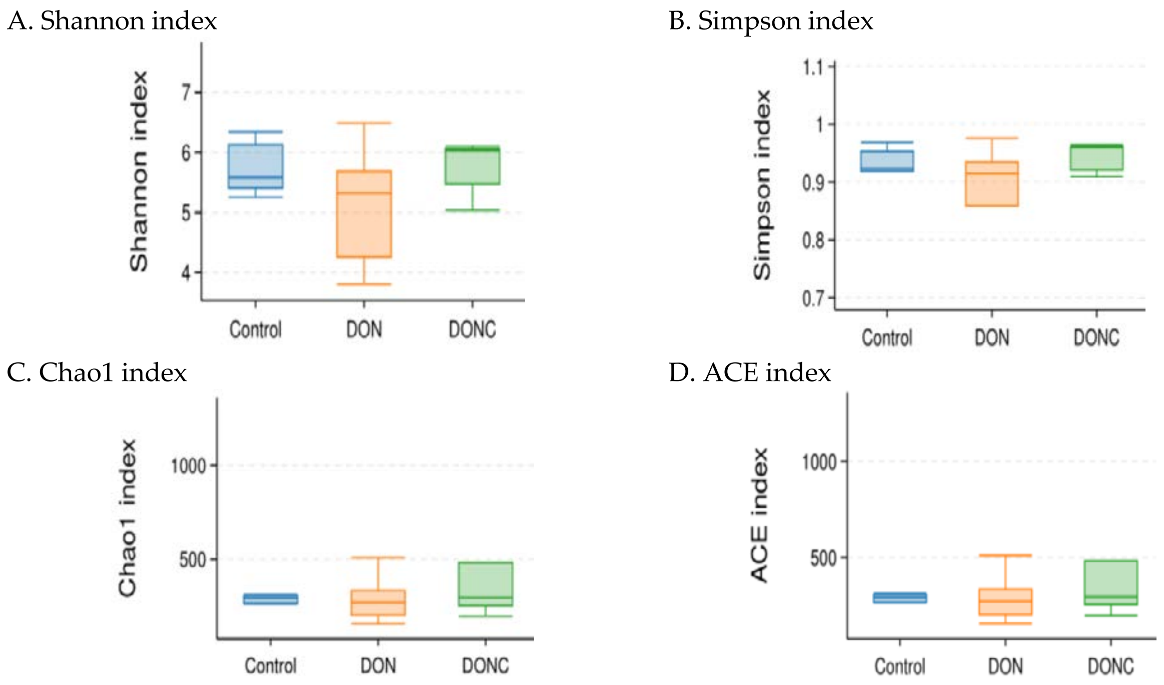

Figure S1. Alpha-diversity analysis of gut microbiota among groups. (A) Shannon index; (B) Simpson index; (C) Chao1 index; (D) ACE index. Control, birds were fed a basal diet; DON, birds were fed a basal diet contaminated with DON; DONC, birds were fed a DON-contaminated basal diet supplemented with 200 U/kg CAT.

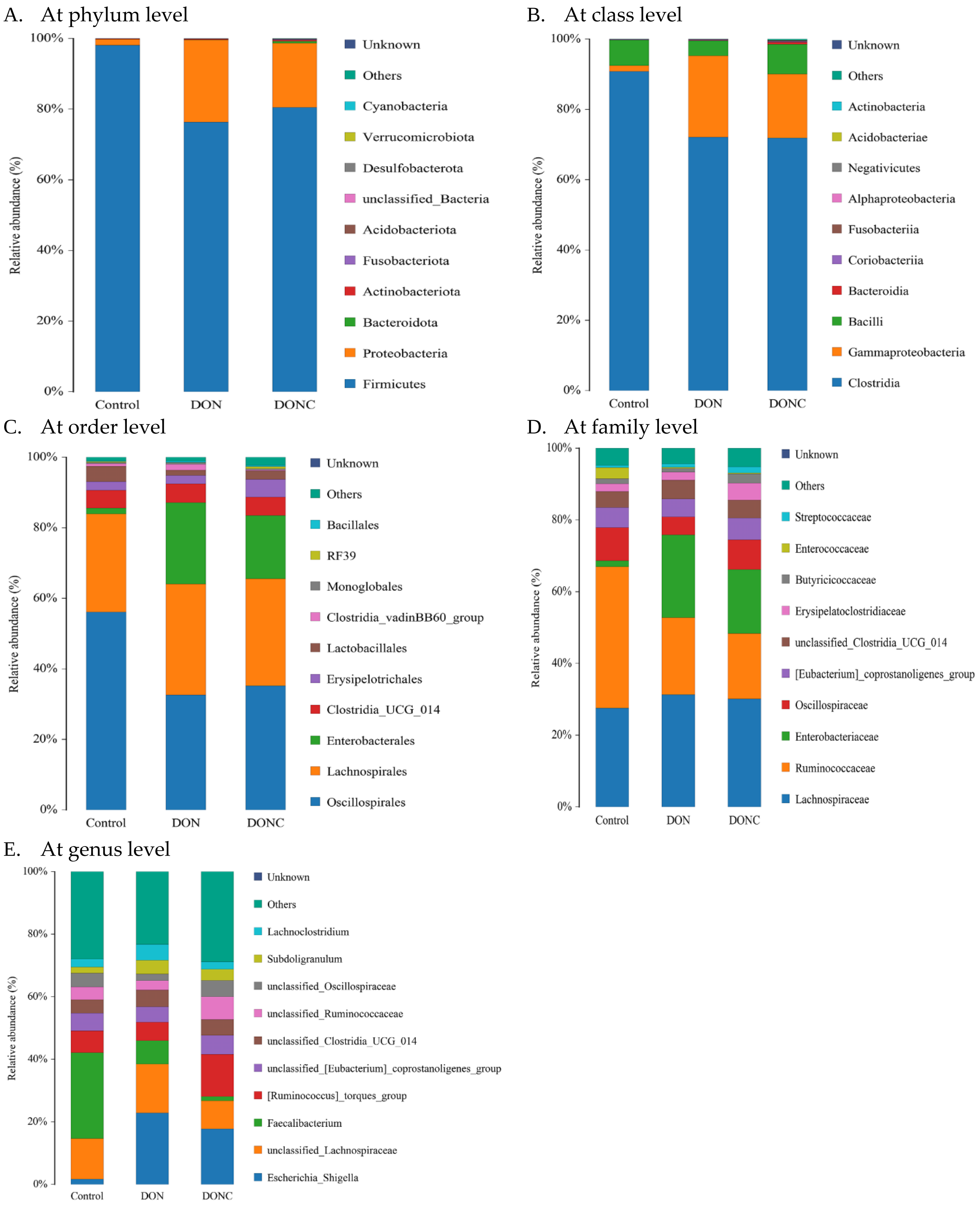

Figure S2. Gut microbial composition of broilers at phylum (A), class (B), order (C), family (D) and genus (E) levels. Control, birds were fed a basal diet; DON, birds were fed a basal diet contaminated with DON; DONC, birds were fed a DON-contaminated basal diet supplemented with 200 U/kg CAT.

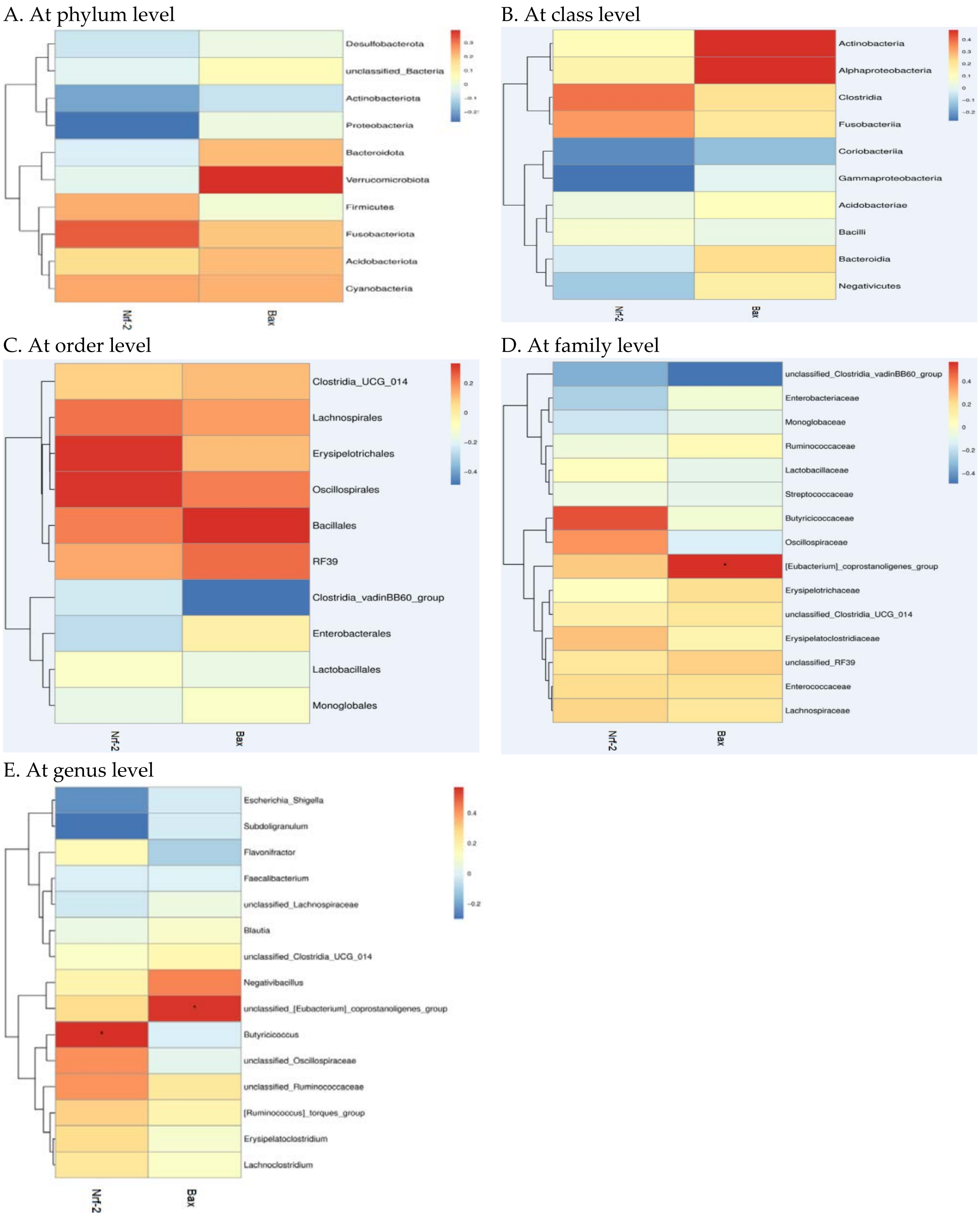

Figure S3. Correlation analysis between gut microbiota (A, at phyum level; B, at class level; C, at order level; D, at family level; E, at genus level) and intestinal gene expression in broilers. Bax, Bcl-2 associated X protein; Nrf2, nuclear factor erythroid-2 related factor. The red and blue panes represent positive and negative correlations, respectively. Color intensity means the Spearman's r-value of correlations in each panel. The asterisks indicate significant correlations ($^{*}p < 0.05$;). Control, birds were fed a basal diet; DON, birds were fed a basal diet contaminated with DON; DONC, birds were fed a DON-contaminated basal diet supplemented with 200 U/kg CAT.
